# The Locally Driven Temporal Bone Dissection Laboratory: A Sustainable Tool for Otologic Development in Sub‐Saharan Africa

**DOI:** 10.1002/oto2.70134

**Published:** 2025-05-23

**Authors:** Nina R. Patel, Fayaz Jaffer, Aveline Kahinga, Shaban Mawala, Mary Jue Xu, Aslam Nkya, Jeffrey Sharon, Eric K. Kim, Sayyeda Datoo‐Jaffer, Stephanie Unterrieder, Ali F. Jaffer

**Affiliations:** ^1^ Otolaryngology–Head and Neck Surgery Department University of California San Francisco San Francisco California USA; ^2^ Global OHNS Initiative Dar es Salaam Tanzania; ^3^ HearWell Audiology Clinic Dar es Salaam Tanzania; ^4^ Department of Otorhinolaryngology Muhimbili University of Health and Allied Sciences Dar es Salaam Tanzania; ^5^ Department of Otorhinolaryngology Muhimbili National Hospital Dar es Salaam Tanzania; ^6^ MED‐EL Medical Electronics Innsbruck Austria

**Keywords:** otology, sub‐Saharan Africa, surgical education, temporal bone

## Abstract

**Objective:**

To evaluate the feasibility of a temporal bone dissection laboratory in Tanzania to support otologic surgical training for otolaryngologists in the region.

**Study Design:**

This prospective cohort study evaluates six temporal bone laboratory training sessions over the course of 11 months. Pretraining and posttraining surveys were distributed during the first year of implementation in 2023. Postsurveys were distributed both immediately and 6 months posttraining.

**Setting:**

Single tertiary care academic medical center in Dar es Salaam, Tanzania.

**Methods:**

Participant data for 47 attendees including country/region of practice, otologic procedures completed, and number of trainees/audiologists in their clinical practice were collected in the pretraining survey. A 5‐point Likert scale was used to assess pretraining and posttraining comfort with completing common otologic procedures. Study outcomes included comfort level, barriers to implementing acquired knowledge, and overall training quality.

**Results:**

The results highlight that participants did not have pretraining comfort with, exposure to, and training with many otologic surgeries. These limitations were largely attributed to barriers identified by participants including limited access to training, learning opportunities, and equipment for otologic procedures. Findings also indicate statistically significant increases in comfort level for the majority of common otologic procedures evaluated.

**Conclusion:**

This study highlights that the training has been both feasible for and desired by participants, and has addressed critical needs in continued surgical training. Temporal bone dissection labs are a feasible and highly desired model to increase the otologic capacity of practicing otolaryngologists regionally and offer a promising approach for addressing the lack of training opportunities in the region.

Globally, more than 1.5 billion people are affected by hearing loss, of which 80% reside in low‐ and middle‐income countries.^1^ In sub‐Saharan Africa, studies have estimated that the prevalence of hearing impairment among adults is 15.7%, much higher compared to 4.9% in high‐income countries.^2^ Significant challenges in access to providers trained in hearing care in addition to screening programs and effective treatment can delay and limit care entirely.

In Tanzania—a country of 64 million people and fewer than 100 otolaryngologists—there have been significant strides in the development of more comprehensive diagnostic services in otology over the past decade.^3^, ^4^ However, as of 2023, there are only three fellowship‐trained otologists in the country. The National Cochlear Implant Program was therefore established in 2013 in partnership with Muhimbili National Hospital (MNH), the largest public and tertiary referral hospital in Tanzania, HearWell Audiology Clinic, and MED‐EL to increase surgical otologic capacity and increase access to cochlear implantation expertise in the country. The first cochlear implant surgeries in Tanzania were undertaken in 2017 by visiting surgeons in collaboration with MED‐EL.

To train local surgeons, MED‐EL and the Austrian Development Agency (ADA) worked with the MNH Department of Otorhinolaryngology to establish the country's first temporal bone dissection lab in February 2023. This lab is intended to provide specialized training to surgeons from sub‐Saharan Africa and build local capacity by providing specialized knowledge and exposure to a range of otology/neurotology training opportunities. Between February and December 2023, six temporal bone courses were run by both visiting and Tanzania‐based instructors. The February 2023 course had 13 participants, the December 2023 course had 11 participants, and the rest all enrolled 12 participants each.

Here, we report results from preintervention and postintervention surveys that aimed to capture the need for the temporal bone dissection laboratory, the impact of the laboratory on training, and the potential for sustainable capacity‐building. By understanding the educational impact of implementing this newly established temporal bone dissection lab, future training regionally can tailor curriculum to regional needs and maximize the benefit of training sessions with the long‐term goal of improving the availability of surgical otologic capacity regionally.

## Methods

### Study Design and Participants

This preintervention and postintervention study involved administering preintervention and postintervention surveys evaluating the exposure, comfort, and training with otologic procedures for participants attending the temporal bone dissection courses. The surveys were completed by all participants who underwent temporal bone dissection training courses over the first year of its establishment in 2023 at MNH in Dar es Salaam, Tanzania. Surveys were administered immediately before and after the training courses.

Each course itself was a 2‐day hands‐on temporal bone dissection workshop. Each participant was assigned one cadaveric temporal bone for dissection under supervision. Didactic sessions covered foundational and advanced topics including temporal bone anatomy, mastoidectomy techniques, ossicular chain reconstruction, and grommet insertion. Faculty‐led demonstrations and practical sessions were used to reinforce learning. Technical and equipment challenges—such as limited access to dissection stations and microscopes—were addressed through partnerships with international collaborators who donated essential equipment. The course was designed to be low‐cost and replicable, with an emphasis on practical experience despite infrastructural limitations.

The participants for the courses were identified and invited specifically by the organizers of the course. Recruitment for the surveys included having all participants complete the presurveys before beginning the training session, and additionally, complete the postsurvey immediately after completing the training session. Despite having 72 participants who attended the training in 2023, only 47 participants were able to complete both the pretraining and posttraining surveys since the pretraining survey was only implemented after the May 2023 course; therefore, this study evaluated surveys of 47 participants. Postsimulation surveys were conducted 6 months after completion of the course and were distributed to all prior participants virtually.

This study was approved by the University of California San Francisco (UCSF) Institutional Review Board (study #24‐41001).

### Data Collection Tools and Procedures

Surveys before the temporal bone dissection courses captured demographic characteristics and baseline clinical access and practice, comfort with otologic procedures, performance of otologic procedures, and access to training and education. Comfort level with otologic procedures was determined on a Likert scale with a predefined scale indicating 1 as not comfortable and 5 being very comfortable. Surveys after the temporal bone dissection courses captured participant acceptability of the training and barriers to implementing the acquired knowledge. These two factors were assessed through open‐ended template sections that allowed participants to share their feedback. Surveys were collected in an electronic format via Google Forms, and links were distributed to participants immediately before and 6 months before the training session.

### Data Analysis

The data were interpreted using a mixed‐methods analysis. Descriptive statistics were used to capture the open‐ended postsurvey data provided regarding respondent acceptability of the temporal bone training course as well as barriers to implementing acquired knowledge. Using thematic analysis, the data were coded using a codebook that defined broader themes captured in the free response data. Other presurvey data were presented with descriptive statistics showcasing quantitative demographic information, performance, comfort, and training with otologic surgeries.

## Results

The study captured a total of 47 survey respondents who participated in the temporal bone dissection training course; detailed information about the respondents can be found in Table 1. Geographically, most attendees are regionally based in East Africa with the prominent majority residing within Tanzania; other regional representations included Ethiopia, Ghana, Kenya, Rwanda, and Uganda. Table 1 also highlights that roughly 44.7% (21/47) of respondents had not completed any otologic procedures in the past month.

**Table 1:** Baseline Clinical Access and Clinical Practice

Variable	N	% of participants
Country/region of practice		
Tanzania	53	73.6
Kenya	7	9.7
Uganda	5	6.9
Rwanda	4	5.5
Ethiopia	2	2.8
Ghana	1	1.4
Otologic surgeries in the past month		
None	21	44.7
1‐3	12	25.5
4‐7	11	23.4
More than 8	3	6.4
Audiologists in your practice		
None	2	4.3
1‐2	20	42.6
3‐4	7	14.9
5+	18	38.3
Number of trainees they work with		
None	12	25.5
Under 10	21	44.7
10‐20	5	10.6
Above 20	9	19.1

**Figure 1:**
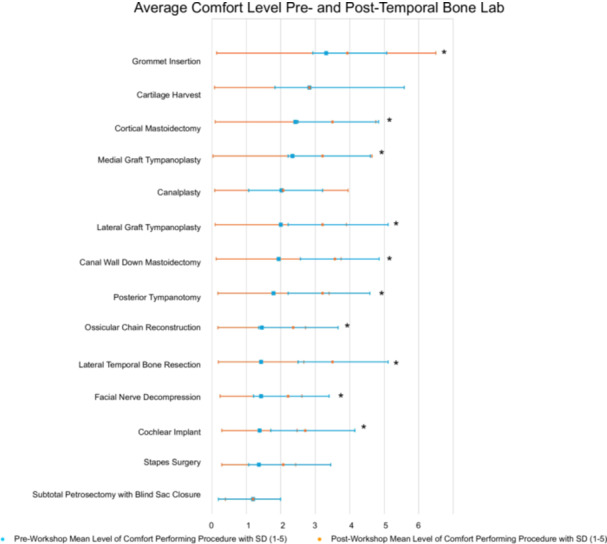
Average comfort level pretemporal and posttemporal bone lab. Average comfort level of participants in performing a number of otologic procedures before and after completion of the temporal bone lab training in Dar es Salaam, Tanzania. Comfort levels were assessed using a standardized 5‐point Likert scale, with higher scores indicating greater confidence. Posttraining scores demonstrate a significant improvement (statistical significance indicated with an asterisk), highlighting the effectiveness of the laboratory in enhancing otologic surgical proficiency.

Participants' previous performance and self‐perceived level of comfort with otologic surgeries before beginning the temporal bone lab are outlined, respectively, in Tables 2 and 3. The three most performed otologic procedures in respondents include grommet insertion at 74.5% (35/47), cortical mastoidectomy at 55.3% (26/47), and cartilage harvesting at 53.2% (25/47). Despite this, on average, the level of comfort for these three procedures preworkshop was 3.32, 2.43, and 2.8, respectively, using a scale of 1 to 5 with 1 being very uncomfortable and 5 being very comfortable. This can be compared to postworkshop comfort levels of 3.39, 3.50, and 2.83. Also of note, only 5% (5/47) of respondents had previously performed a cochlear implant, 42.6% (20/47) had performed a medial graft tympanoplasty, and 27.7% (13/47) had performed a lateral graft tympanoplasty (Table 2). For comparison, on average, the level of comfort for these procedures preworkshop compared to postworkshop was 1.38 versus 2.71, 2.34 versus 3.21, and 2.00 versus 3.21, respectively (Figure 1). Additionally, there was a statistically significant change preworkshop and postworkshop in self‐reported comfort level for the following procedures: grommet insertion, cortical mastoidectomy, medial graft tympanoplasty, lateral graft tympanoplasty, canal wall down mastoidectomy, posterior tympanotomy, ossicular chain reconstruction, lateral temporal bone resection, facial nerve decompression, and cochlear implant.

**Table 2:** Experience Before Temporal Bone Dissection Training

Procedures	Number of providers who have performed procedures	Percentage of providers who have performed procedures
Grommet insertion	35	74.5
Cartilage harvest	25	53.2
Cortical mastoidectomy	26	55.3
Medial graft tympanoplasty	20	42.6
Canalplasty	12	25.5
Lateral graft tympanoplasty	13	27.7
Canal wall down mastoidectomy	14	29.8
Posterior tympanotomy	11	23.4
Ossicular chain reconstruction	7	14.9
Lateral temporal bone resection	2	4.3
Facial nerve decompression	4	8.5
Cochlear implant	5	10.6
Stapes surgery	3	6.4
Subtotal petrosectomy with blind sac closure	1	2.1

**Table 3:** Access to Education and Training

	N	Percentage of prior courses
Prior trainings/temporal bone dissection courses		
None	16	34
One	11	23.4
Two	4	8.5
Three	8	17
More than three	8	17
Requested future training sessions		
Mastoidectomy	21	44.7
Tympanoplasty	16	34
Cochlear Implant	15	31.9
Ossicular chain reconstruction	11	23.4
Facial nerve decompression	6	12.8
Canalplasty	5	10.6
Grommet insertion	3	6.4
Temporal bone resection	3	6.4
Tympanostomy	2	2.3
Internal auditory canal access	1	2.1
Labyrinthectomy	1	2.1
Lateral skull base	1	2.1
Petrosectomy	1	2.1
Stapes	1	2.1

Table 3 outlines the participants' access to education and training at the local, regional, and national level. As shown, the majority of respondents 34% had not participated in previous training sessions or temporal bone dissection courses. Additionally, participants most highly expressed interest in learning/practicing the following procedures: mastoidectomy 44.7% (21/47), tympanoplasty 34% (16/47), and cochlear implant 31.9% (15/47), amongst other procedures outlined in the table.

Participants' reception of the temporal bone dissection training course was also evaluated and captured immediately postsurvey and at 6 months postsurvey. The immediate postsurvey data highlighted that the primary goal for participants attending the session was skills acquisition (21/47) and increased confidence in the procedure (18/47). One respondent stated that their goal was “to be competent in otological surgeries for they are less performed in our settings,” whereas another stated it was to “obtain more competence to build on the previously attended workshops and eventually perform otologic surgeries at my hospital.”

In a follow‐up survey conducted 6 months after completing the training course, 71.4% (10/14) of previous participants agreed that the training sessions met their expectations, and 78.6% (11/14) also stated that they would be able to apply the knowledge they learned to clinical practice. Additionally, 50% (7/14) of the participants reported performing more otologic procedures since attending the training workshop with 87.5% of participants (7/8) stating they have performed myringotomy and grommet insertion, 62.5% participants (5/8) have performed tympanoplasty, 50% (4/8) have performed mastoidectomy, and 12.5% (1/8) have performed cholesteatoma removal in clinical practice. No participants reported performing ossicular chain reconstruction or facial nerve decompression since completing the training. Participants reported interest in attending future training sessions on topics related to the following: live surgeries (71.4%, 10/14), independent drilling session in lab (7/14, 50%), guided supervised drilling session (42.9%, 6/14), and more local temporal bone training (35.7%, 5/14). In summary, 71.4% of 6‐month posttraining participants rated the training as a 4 (excellent) or 5 (good).

Finally, the study sought to recognize the barriers in implementing the skill sets acquired at the temporal bone dissection training courses (Table 4). The most prevalent barrier described by participants after completing the training session is access to equipment (24/47), followed by comfort with procedures (14/47), and access to learning opportunities and knowledge (14/47).

**Table 4:** Barriers to Implementing Acquired Knowledge

Items	n (%)	Definition	Relevant quote
Access to equipment	24 (36.4%)	Lack of regular availability and affordability of a range of surgical instruments required for otologic procedures that are not broken and fully functional	“Infrastructure problems: Break down and lack of equipment”
Access to learning opportunity and knowledge	14 (21.2%)	Lack of availability and accessibility of regular, diverse experiences for practicing skills and formal sharing of expertise in techniques regarding surgical/otologic procedures	“Inadequate trainers and training time”
Comfort with procedures	14 (21.2%)	Lack of confidence in performing and completing respective otologic surgeries	“Lack of advanced ear knowledge [and] expertise”
Access to facilities	7 (10.6%)	Lack of physical space within the hospital that is available for completing otologic surgeries, conducting outpatient visits and follow‐ups, and training sessions for providers and trainees	“Lack of OR space and time”
Access to staff	7 (10.6%)	Lack of available support staff in the hospital for completing surgeries, identifying patients in need, and managing regular follow‐up for hearing health and speech therapy	“Human resources [lacking] and no dedicated audiologist and speech pathologist”

In a follow‐up survey completed 6 months after completing the workshop, participants indicated that the primary barriers to implementing the training included lack of comfort working with live patients (85.7%, 12/14), lack of otology surgical instruments (57.1%, 8/14), lack of drill unit and burs (50%, 7/14), and lack of appropriate microscope (42.9%, 6/14).

## Discussion

This study underscores a critical need for otologic training among participants of the temporal bone dissection course, as evidenced by low reported comfort levels across both simple and complex procedures. Even for relatively common surgeries such as grommet insertion and cartilage harvest, participants expressed limited confidence. The near‐universal discomfort—despite the high regional burden of chronic ear disease—highlights a significant training gap.

As demonstrated, regional and national temporal bone dissection labs are both feasible and well‐received. Participants reported increased knowledge and improved comfort following the course, reinforcing the role of simulation‐based training in strengthening surgical capacity. However, training alone is insufficient. Infrastructure limitations—such as lack of microscopes, surgical equipment, and reliable operating facilities—remain major barriers. Nearly 45% of participants reported performing no otologic surgeries in the past month, despite the clear clinical need. This discrepancy emphasizes that education must be paired with resource investment to translate training into practice.

Our approach builds on a growing body of literature examining surgical training in LMICs, such as the Surgical Management and Reconstructive Training (SMART) course for orthopedic reconstruction.^5^ Similar to SMART, our study found that travel costs and course affordability are major barriers. Unlike previous work, however, our initiative is rooted within the region and tailored to otolaryngology—a specialty that has seen less focus in global surgical training. By conducting the course in Tanzania and serving neighboring countries, we provide a more cost‐effective and sustainable model. Notably, training 12 surgeons locally costs the same as sending a single surgeon abroad.

Investing in regional programs fosters broader capacity‐building. The “train‐the‐trainer” model—supported by both simulation tools and online learning—has the potential to create a ripple effect in education and service provision. Our early results suggest that this approach can improve surgical confidence, particularly for essential procedures like tympanoplasties and mastoidectomies, and gradually prepare surgeons for more advanced surgeries such as cochlear implantation and facial nerve decompression.

Although early results are promising, our findings are limited by the short duration of implementation and the scope of the postcourse evaluation. Future sessions will include expanded follow‐up and more robust assessment tools to better evaluate long‐term outcomes. Additionally, ongoing curriculum refinement will ensure the training meets evolving learner needs.

In conclusion, this pilot highlights both the urgent demand for otologic training and the systemic barriers that limit surgical care in sub‐Saharan Africa. By embedding training within the region, leveraging local institutions, and addressing both educational and infrastructural needs, we present a replicable model to improve otologic care and reduce the burden of ear disease across the region.

## Conclusion

Since its opening in February 2023, the temporal bone dissection laboratory in Dar es Salaam has hosted multiple hands‐on training sessions, drawing participants from across sub‐Saharan Africa. Quantitative and qualitative feedback from attendees indicates a measurable increase in comfort and confidence with otologic procedures following the training. These findings support the feasibility of implementing regionally based surgical training models in low‐resource settings and underscore the lab's potential as a sustainable platform for strengthening otolaryngology capacity in the region.

## Author Contributions

**Nina R. Patel**, conceptualization, validation, formal analysis, data curation, investigation, writing; **Fayaz Jaffer**, administration, review and editing, supervision; **Aveline Kahinga**, supervision, review and editing, validation; **Shaban Mawala**, supervision, review and editing; **Mary Jue Xu**, conceptualization, review and editing, supervision; **Aslam Nkya**, supervision, review and editing, administration, resources; **Jeffrey Sharon**, conceptualization, resources; **Eric K. Kim**, methodology, conceptualization, review and editing; **Sayyeda Datoo‐Jaffer**, formal analysis, data curation, review and editing; **Stephanie Unterrieder**, supervision, project administration, resources, review and editing; **Ali F. Jaffer**, conceptualization, methodology, investigation, data curation, writing, supervision.

## Disclosures

### Competing interests

None.

### Funding source

None.
